# Stimulus Prediction and Postural Reaction: Phase-Specific Modulation of Soleus H-Reflexes Is Related to Changes in Joint Kinematics and Segmental Strategy in Perturbed Upright Stance

**DOI:** 10.3389/fnint.2018.00062

**Published:** 2018-12-19

**Authors:** Ramona Ritzmann, Kyungsoo Lee, Anne Krause, Albert Gollhofer, Kathrin Freyler

**Affiliations:** ^1^Department of Sport and Sport Science, University of Freiburg, Freiburg, Germany; ^2^Institute of Training and Computer Science, German Sport University Cologne, Cologne, Germany

**Keywords:** perturbation, anticipation, electromyography, H-reflex, neuromuscular, prediction, kinematics

## Abstract

Anticipation determines the timing and efficiency of human motor performance. This study aimed to evaluate the effect of stimulus anticipation on proactive (prior to the event) and reactive (after the event) postural adjustments in response to perturbations. Postural set was manipulated by providing either (i) predictable, (ii) unpredictable, or (iii) cheated perturbations which require balance corrections to maintain postural stability. In 29 subjects, a protocol of anterior and posterior perturbations was applied for the conditions (i–iii). Center of pressure (COP) displacement, ankle, knee, and hip joint kinematics and electromyographic activity (EMG) of the soleus (SOL) and tibialis anterior (TA) muscles were recorded prior (PRE) and after posterior perturbations. SOL H-reflexes at the peak of the short-, medium- ,and long-latency responses (SLR, MLR, LLR) were assessed. For conditions (i to iii) EMG activity and COP differed prior to perturbation onset (*p* < 0.05). After perturbation, results demonstrated a progressively increased H-reflex amplitude in the MLR and LLR (*p* < 0.05), delayed muscle activities (*p* < 0.05), and shifted activation patterns, with muscles of the proximal segment being more involved in the compensatory postural response (*p* < 0.05). COP displacements and ankle, knee, and hip joint deflections progressively increased (*p* < 0.05). Neuromechanical coupling showed positive correlations for the anticipation-induced changes in EMG activity and H-reflex amplitude with that of COP displacement (*p* < 0.05). In conclusion, proactive and reactive postural responses indicated setting dependent modulations of segmental and phasic muscle activation. A shift to proximal muscle groups and facilitated late reflex responses compensating for cheated or unpredicted perturbations was found to recover a safe body equilibrium. In consideration of the phase-specific adaptation and its interrelationship to the kinematics, it suggested that changes in stimulus prediction challenged the central nervous system to appropriately counteract the higher postural challenges. The outcomes of this experiment are of functional relevance for experimental and training settings involving perturbation stimuli. These findings provide fundamental information of the mechanisms underlying postural adjustments in response to external perturbations.

## Introduction

Humans experience perturbations applied to their body (Macpherson et al., 1989) due to the displacement of the body's center of mass (COM) beyond the boundaries of the base of support (Maki and McIlroy, 1996). The central nervous system (CNS) uses 2 main strategies to restore balance if it is disrupted by a perturbation: (1) the proactive postural adjustments based on feed-forward mechanisms made throughout the anticipation phase at a conscious and subconscious level *prior* to perturbations (Belen'kii et al., 1967; Mohapatra et al., 2012), and (2) the compensatory postural adjustments based on feedback initiated by sensory signals *after* perturbations (Horak and Nashner, 1986; Horak et al., 1989; Mohapatra et al., 2012). Whereas proactive adjustments serve to minimize the displacement of the body's COM beforehand (Bouisset and Zattara, 1987), compensatory postural adjustments serve to reposition the COM after a perturbation that has already occurred using Ia afferent reflex circuitry coupled with other CNS pathways (Macpherson et al., 1989; Maki and McIlroy, 1996). Studies demonstrate that subjects exposed to perturbation show robust stereotyped compensatory patterns of leg muscle activity which are phase- and segment-specific with regard to both the movement direction and the magnitude of surface displacement (Cresswell et al., 1994; Freyler et al., 2015).

Recent research suggests that the predictability of the perturbation stimulus can alter the recovery strategies and modify postural adjustments breaking the robust stereotyped neural pattern (Horak et al., 1989; Taube et al., 2008). Knowledge about whether the perturbation will occur changes the timing and magnitude of postural reactions and determines the recovery success in restoring equilibrium (Horak et al., 1989; Kourtis et al., 2008). In terms of anticipation, the predictability has been clustered in three modalities based on an increased level of difficulty: (i) predictable, (ii) unpredictable, and (iii) wrongly predicted perturbation of postural stability (Horak et al., 1989). The latter modality “wrongly predicted” has been described in several contexts as a cheat condition, being the most difficult to manage among all perturbations (Horak et al., 1989). The neuro-mechanics of the recovery response in regard to the predictability of the perturbation, whether (i, ii, or iii), is the topic of the current paper. Despite articles comparing these three modalities being rare, the state of the art in terms of neuromuscular and kinematic distinctions is described below.

First, when the perturbation stimulus is known, stimulus characteristics are anticipated and proactive postural adjustments such as muscle activation and joint positioning is preconfigured by the supraspinal structures of the CNS (Belen'kii et al., 1967; Bouisset and Zattara, 1987). As a consequence, postural disturbance can be minimized (Belen'kii et al., 1967).

Second, when compensating for an unpredictable stimulus, proactive postural adjustments are absent and reactive compensatory responses predominate. Studies show a gradually increased sway path (Jacobs et al., 2008; Santos et al., 2010; Kanekar and Aruin, 2014a,b), augmented magnitude, timing differences (Aimola et al., 2014), variability of the postural response (Okai and Fujiwara, 2013; Kanekar and Aruin, 2014a,b), modified sensory processing (Kourtis et al., 2008), changes in neuromuscular activation reflected by delayed neuromuscular responses (McChesney et al., 1996), and modulated spinal (Burleigh-Jacobs et al., 1997) and supraspinal contributions (Adkin et al., 2006; Jacobs et al., 2008), which are also related to an increased fall and injury incidence (Mawston et al., 2007; Gehring et al., 2014; Pater et al., 2015). Furthermore, a shift in balance strategy could be observed. Unpredictable compared to predictable perturbations are associated with a gradual reduction in ankle strategy and concomitantly increased hip strategy (Mawston et al., 2007; Okai and Fujiwara, 2013; Mani et al., 2014).

Third, neuromuscular activation is disturbed in events with wrong expectations, i.e., smaller or bigger although allocated vice versa. In such cheating conditions, the activation of the musculature is pre-programmed to minimize balance disturbances with a subsequent overestimation of the response when a larger or faster perturbation was expected and underestimated response when a smaller or slower perturbation was expected in contrast to the actual stimulus event (Horak et al., 1989).

Unlike perturbation amplitude and velocity, the consequences of wrongly predicted perturbation directions have not been studied; clearly, agonists and antagonists are affected inversely with respect to the perturbation. However, little is known about the changes in spinal excitability modulating Ia afferent transmission, although reflexes are reported to be of major relevance for the reactive control of posture (Horak and Nashner, 1986; Gollhofer and Rapp, 1993). From studies examining compensatory neuromuscular responses after perturbations disregarding stimulus anticipation, it is known that muscular activation patterns are characterized by phase-specific reflex components indicated as short- (SLR), medium- (MLR) and long-latency responses (LLR) following the onset of perturbation (Horak and Nashner, 1986; Dietz et al., 1991; Gollhofer and Rapp, 1993). In particular, Gollhofer et al. (1989) demonstrated that functionally relevant muscle activation (>65 ms after onset, MLR and LLR) serves to relocate the COM back to the vertical. MLR and LLR are supposed to be attributed to spinal, polysynaptic reflexes, and have functional significance in inducing appropriate active joint moments for the preservation of postural stability (Horak and Nashner, 1986; Gollhofer et al., 1989; Horak et al., 1989). Slight postural disturbances are compensated by immediate, non-functional monosynaptic stretch responses in the SLR (Gollhofer et al., 1989; Gollhofer and Rapp, 1993).

Despite the widespread relevance of stimulus prediction in different areas of the rehabilitative medicine and geriatrics, as well as a substantial number of related articles (McChesney et al., 1996; Kourtis et al., 2008; Okai and Fujiwara, 2013; Kanekar and Aruin, 2014a,b), the underlying neuromuscular mechanisms in terms of posture control are poorly understood. No study has analyzed the interrelation of neuromuscular and kinematic modulations in terms of the three modalities of prediction.

**Figure 1:**
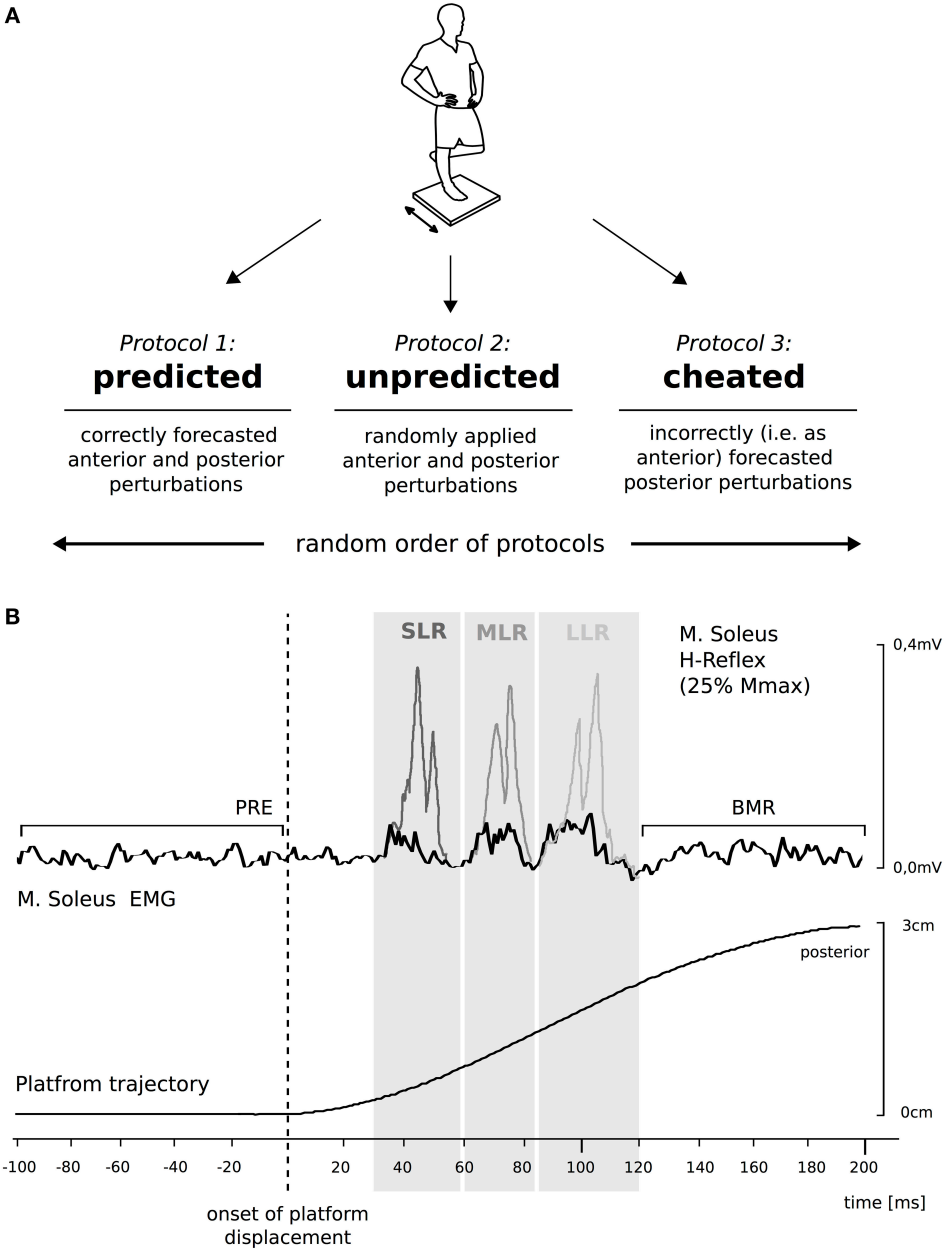
Protocols to establish the effect of anticipation on compensatory postural responses to perturbations. **(A)** The scheme on the top displays the experimental design. Anticipation was differentiated by the forecast of direction: either the direction was correctly predicted (predicted), occurred randomly (unpredicted), or was wrongly predicted (cheated). The graph at the bottom **(B)** illustrates the mean trajectory of 10 posterior surface displacements during perturbed stance; time zero corresponds to the onset of perturbation. The Soleus (SOL) background electromyogram (EMG) (black line) of one representative subject comprising the mean of 10 trials is displayed. SOL H-reflexes (gray lines) were timed to coincide with the peaks of short- (SLR, dark gray graph), medium- (MLR, gray graph), and long-latency responses (LLR, light gray graph). Background EMG, H-reflexes, belated muscle responses (BMR) and M-waves (not illustrated) were compared between the three protocols by analyzing the integrals below the curves in the respective time intervals (gray boxes).

Therefore, we aimed in this study to evaluate the effect of stimulus prediction on body equilibrium, neuromuscular control, spinal excitability, and joint kinematics in response to perturbation. Three protocols were used (Figure 1A): (1) perturbation direction was correctly predicted using auditory pre-cueing; (2) perturbation direction was unpredicted and allocated randomly and thus, unknown to the subject; and (3) subjects were cheated in regard to the direction. We were particularly interested in identifying if and how stimulus prediction was counterbalanced at a neuromuscular and kinematic level, and if this neuro-mechanical coupling could be attributed to particular reflex phases, spinal transmissibility of the Ia afferent circuitry or body segments. We hypothesized that modulations in response to stimulus prediction would be phase- (SLR, MLR, and LLR) and segment-specific (distal and proximal), and might be associated with a shift in the balance strategy accompanied by differences in kinematic output. Thus, from predicted to unpredicted to cheated perturbations we expect a delayed recovery response from the immediate to the late reflex phases concomitant with a gradual shift from the distal to the proximal limb segments. Furthermore, we predicted that the two principal mechanisms that the CNS uses to maintain equilibrium while standing (Santos et al., 2010), the proactive (prior to stimulus) and the reactive (after the stimulus) postural adjustments, would differ considerably for the cheat condition when error detection was required.

## Materials and Methods

### Subjects

Twenty-nine subjects (14 females, 15 males, age 26 ± 3 years, weight 71 ± 6 kg; height 172 ± 8 cm; values expressed as mean ± standard deviation) volunteered to participate in this study. Eligibility criteria were general good health and no previous neurological irregularities or injuries of the lower extremities. Exclusion criteria were pregnancy, illness, injuries, vestibular, or proprioceptive dysfunction, previous surgeries on the left or right leg, neuro-degenerative diseases or episodes associated with neural dysfunction and an age >35 years. All subjects gave written informed consent to the experimental procedure, which was approved by the ethics committee of the University of Freiburg (EK Freiburg: 15/13), and was in accordance with the latest revision of the Declaration of Helsinki. A priori, the sample size was estimated by means of a power analysis based on a previously executed pilot study (*f* = 0.90; alpha = 0.05; power = 0.90).

### Experimental Design

A single-group repeated-measures study design was used in order to evaluate the effect of stimulus anticipation of perturbation direction on anticipatory adjustments, reflex responses, center of pressure (COP) displacement, joint kinematics, and segmental organization for a perturbed unilateral stance. To identify changes in spinal excitability, Soleus (SOL) muscle H-reflexes were applied during posterior perturbations at the peak of the SLR, MLR, and LLR, respectively. Single transient horizontal perturbations to the support surface were applied to the subject in anterior or posterior direction using Perturmed® (Brüderlin, Göppingen, Germany; Freyler et al., 2015).

Subjects stood barefoot on their right leg with the left leg flexed at an angle of 90°, with the knees touching. Perturbations were applied in an anterior or posterior direction at intervals of 4–8 s, with an average amplitude of 3 cm and duration of 210 ms (Freyler et al., 2015). The timing and magnitude of the perturbation was controlled using a potentiometer attached to the support surface in order to trace the platform's trajectories (Figure 1; means ± standard deviations are displayed in Table 1). One experimenter situated on the subject's left side provided assistance, if necessary, to avoid a fall. Measurements were taken over periods consisting of consecutive 30 s exposures to perturbation with intervals of 30 s rest (Lesinski et al., 2015).

**Table 1:** Kinematics.

**Parameter**		**Protocols**			**Statistics**	
	**PHASE**	**Predicted**	**Unpredicted**	**Cheated**	***rmANOVA interaction***	***rm*ANOVA**
**PLATFORM MOVEMENT**						
Perturbation amplitude [Δcm]	Peak	3 ± 0.0	3 ± 0.0	3 ± 0.0	–	*P* = 0.981; *F* = 0.01; $\eta_{\text{p}}^{2}$ = 0.02
Perturbation duration [ms]	Peak	211 ± 4	209 ± 3	211 ± 4	–	*P* = 0.853; *F* = 0.04; $\eta_{\text{p}}^{2}$ = 0.03
Mean velocity [m*s^−1^]	Peak	1.8 ± 0.1	1.8 ± 0.1	1.8 ± 0.1	–	*P* = 0.720; *F* = 0.03; $\eta_{\text{p}}^{2}$ = 0.01
Peak acceleration [m*s^−2^]	Peak	12.6 ± 0.9	12.4 ± 0.8	12.4 ± 0.9	–	*P* = 0.906; *F* = 0.03; $\eta_{\text{p}}^{2}$ = 0.01
**COP DISPLACMENT**						
COP_onset_ [cm]	Onset	**−0.7 ± 0.5**	−0.1 ± 0.1*	0.7 ± 0.2*	**P < 0.001**	***P*** **< 0.001;** ***F*** **= 12.1;** $\eta_{\text{p}}^{2}$ **= 0.19**
COP_90ms_ [cm]	**90 ms**	**−1.4 ± 0.5**	−1.5 ± 0.6*	−1.1 ± 0.4*	**F = 15.7**	***P*****< 0.001;** ***F*** **= 34.9;** $\eta_{\text{p}}^{2}$ **= 0.37**
COP_120ms_ [cm]	**120 ms**	**−2.1 ± 0.9**	**−2.5 ± 0.7**	−2.5 ± 0.6*		***P***<**0.001;** ***F*** **= 4.9;** $\eta_{\text{p}}^{2}$ **= 0.17**
COP_peak_ [cm]	**Peak**	**−2.5 ± 0.7**	−3.1 ± 0.9*	−3.7 ± 1.1*		***P*** **= 0.006;** ***F*** **= 8.5;** $\eta_{\text{p}}^{2}$ **= 0.13**
COP peak index [ms]	Peak	230 ± 44	287 ± 65*	344 ± 73*		***P*** **< 0.001;** ***F*** **= 30.1;** $\eta_{\text{p}}^{2}$ **= 0.32**
**JOINT DEFLECTIONS**						
Hip joint position onset [°]	Onset	179.0 ± 6.7	179.1 ± 5.9	179.5 ± 4.8	**P < 0.001**	*P* = 0.463; *F* = 0.8; $\eta_{\text{p}}^{2}$ = 0.05
Hip joint excursion [Δ°]	**Peak**	**1.2 ± 0.9**	**4.6 ± 2.6**	6.1 ± 2.7*	**F = 17.6**	***P*** **< 0.001;** ***F*** **= 48.2;** $\eta_{\text{p}}^{2}$ **= 0.56**
Peak index [ms]	**Peak**	**225 ± 95**	278 ± 89*	346 ± 120*		***P*** **= 0.011;** ***F*** **= 15.7;** $\eta_{\text{p}}^{2}$ **= 0.27**
Knee joint position onset [°]	Onset	178.3 ± 2.9	178.6 ± 3.0	178.9 ± 3.0	**P = 0.001**	*P* = 0.76; *F* = 0.3; $\eta_{\text{p}}^{2}$ = 0.03
Knee joint excursion [Δ°]	**Peak**	**1.5 ± 1.2**	**4.1 ± 2.8**	**5.9 ± 3.0**	**F = 8.7**	***P*** **< 0.001;** ***F*** **= 16.6;** $\eta_{\text{p}}^{2}$ **= 0.22**
Peak index [ms]	**Peak**	**230 ± 109**	**268 ± 107**	290 ± 111*		***P*** **< 0.001;** ***F*** **= 26.1;** $\eta_{\text{p}}^{2}$ **= 0.38**
Ankle joint position onset [°]	Onset	**96.4 ± 4.4**	**95.9 ± 4.5**	**95.0 ± 4.2**	**P = 0.002**	***P*** **= 0.09;** ***F*** **= 7.7;** $\eta_{\text{p}}^{2}$ **= 0.15**
Ankle joint excursion [Δ°]	**Peak**	**2.0 ± 0.5**	**4.7 ± 2.3**	**7.1 ± 2.8**	**F = 7.2**	***P*** **< 0.001;** ***F*** **= 28.6;** $\eta_{\text{p}}^{2}$ **= 0.38**
Peak index [ms]	**Peak**	**192 ± 69**	**216 ± 77**	227 ± 85*		***P*** **= 0.009;** ***F*** **= 24.9;** $\eta_{\text{p}}^{2}$ **= 0.29**

The top section of the table displays the perturbation physics. The middle section shows the center of pressure (COP) displacement for the protocols predicted, unpredicted, and cheated at onset, and 90 and 150 ms after the onset of perturbation; peak displacement and the corresponding index are also recorded. The bottom section displays the hip, knee, and ankle joint position at onset and peak deflection and its corresponding index. Data are displayed for the predicted, unpredicted and cheated protocols. P and F values denote the level of significance of phase x anticipation interaction effects. Bold letters represent a significant effect of anticipation for the rmANOVA. ^*^indicates significant differences compared to the predicted condition.

### Protocols

Three different protocols were used applying anterior/posterior surface translations (Figure 1A): (i) the perturbation direction was known and thus predictable, this protocol served as a control; (ii) aimed to assess perturbation-induced effects when unpredictable perturbations occurred randomly in an anterior or posterior direction and the direction of deterioration stimulus was unknown (Okai and Fujiwara, 2013; Kanekar and Aruin, 2014a,b); and (iii) we investigated the influence of the perturbation when subjects were cheated and the direction was indicated incorrectly. Protocols were executed in a random order by using sealed envelopes, though operators were not blinded. Data were collected at the Institute of Sport and Sport Science, the University of Freiburg, Germany.

#### Protocol 1: Predicted–Perturbation Direction Was Correctly Forecasted

Surface translations were applied randomly in an anterior (#40) or in a posterior (#40) direction. For each trial, we announced the perturbation direction (anterior or posterior) acoustically 1 s prior to the triggered platform movement. For posterior direction, 10 repetitions were used to assess spinal excitability in the SLR, 10 repetitions for MLR, 10 repetitions for LLR, and 10 repetitions without stimulation. H-reflex stimulations were applied randomly.

#### Protocol 2: Unpredicted–Perturbation Direction Was Unknown and Not Forecasted

Eighty surface translations were applied randomly in an anterior (#40) or in a posterior (#40) direction. Perturbation direction was unknown. For posterior direction, 10 repetitions were used to assess spinal excitability by means of H-reflexes in the SLR, 10 repetitions for MLR, 10 repetitions for LLR, and 10 repetitions without stimulation. H-reflex stimulations were applied randomly.

#### Protocol 3: Cheated–Perturbation Direction Was Wrongly Indicated

Perturbation direction was announced acoustically in an anterior direction for each single trial, 1 s prior to platform movement, although subjects were cheated in 1 out of 15 repetitions. In the cheated trials, platform movement occurred in a posterior direction (#40). Surface translations were allocated randomly. For cheated posterior direction, 10 repetitions were used to assess spinal excitability in the SLR, 10 repetitions for MLR, 10 repetitions for LLR, and 10 repetitions without stimulation. H-reflex stimulations were applied randomly; we applied 600 perturbations.

### Outcome Measures

COP displacement, joint goniometry, electromyograms (EMG), and H-reflexes were recorded synchronously using LabVIEW (National Instruments, Texas, United States). As a reference for the COP trajectories and joint kinematics, 1 leg stance without perturbation was recorded for 10 s.

#### Postural Sway

Postural sway was quantified by means of a pressure distribution measuring system (Pedar®, Novel, Germany). The sensor mat was placed upon the perturbation platform; the COP was recorded by means of 3D sensor deformation with a 100 Hz sampling rate and a spatial resolution of 4 sensors per square centimeter. COP displacement was assessed in an anterior and posterior direction and averaged over the trials for each subject and each of the conditions (Cabeza-Ruiz et al., 2011). In addition, COP starting position was assessed at the onset of surface translations to establish initial posture.

#### Joint Goniometry

Ankle (dorsiflexion and plantar flexion), knee (flexion and extension), and hip (flexion and extension) joint kinematics in the sagittal plane were recorded during posterior perturbation with electrogoniometers (Biometrics®, Gwent, United Kingdom). Goniometers were fixed at the joints according to Freyler et al. (2015). All signals were recorded with a sampling frequency of 1 kHz. In addition, joint starting position was assessed at the onset of surface translations to establish initial posture.

#### EMG Recording

Bipolar Ag/AgCl surface electrodes (Ambu Blue Sensor P, Ballerup, Denmark; diameter 9 mm, center-to-center distance 25 mm) were placed over the SOL, the medial gastrocnemius (MG), tibialis anterior (TA), rectus femoris (RF), biceps femoris (BF), and gluteus maximus (GMax) muscles of the right leg. The longitudinal axes of the electrodes were attached in parallel with the underlying muscle fibers. The reference electrode was placed on the patella. Inter-electrode resistance was kept below 2.5 kΩ by means of shaving, light abrasion, degreasing, and disinfection of the skin. The EMG signals were transmitted to an amplifier (band-pass filter 10 to 1 kHz, 1,000 × amplified) via shielded cables and recorded with 1 kHz. The cables were carefully taped to the skin.

For normalization of the EMG data, prior to the measurements subjects performed 3 isometric maximal voluntary contractions (MVC) for each muscle to be recorded; we used the trial with the highest EMG for data normalization with an interval containing the 50 ms prior to and the 50 ms after the maximal amplitude. The MVCs executed as previously published (Wiley and Damiano, 1998; Roelants et al., 2006) were performed isometrically against resistance and held for 3 s. Between trials and repetitions, subjects had recovery pauses of 1 min. Body position during MVCs was strictly controlled and standardized through supervision by the authors and from goniometric recordings of ankle, knee, and hip joint angles (Wiley and Damiano, 1998; Roelants et al., 2006). Antagonistic muscle activation was monitored and trials were repeated when antagonists were activated.

#### H-Reflexes

Anticipation-induced modulation in Ia afferent transmission of the SOL motoneuron pool in protocols i–iii was assessed by H-reflex measurements. H-reflexes were elicited by peripheral nerve stimulation (PNS) with single rectangular pulses of 1 ms duration (Digitimer DS7, Digitimer, Welwyn Garden City, United kingdom). The anode (10 × 5 cm dispersal pad) was fixed directly below the patella on the anterior aspect of the knee. The cathode (2 cm in diameter) was placed in the popliteal fossa. Its location was modified until the best position was found for eliciting a reliable biphasic H-reflex in the SOL. H-reflexes were elicited by electrically stimulating the posterior tibial nerve. Based on recorded H-reflex/M-wave (H/M) recruitment curves obtained during the preparation for this study (data not shown), we defined the stimulation current for the measurements (Crone and Nielsen, 1989). The stimulation current was set constantly to elicit H-reflex amplitudes equal to 25% of the maximal M-wave for quiet one leg stance (Crone and Nielsen, 1989; Taube et al., 2008). For data collection, PNS was triggered to occur in SOL at the peak of the SLR, MLR, or LLR, respectively, during posterior perturbation (Taube et al., 2007). For that purpose, 20 posterior platform displacements were made prior to measurements for each of the subjects in order to define the individual reflex peaks, as illustrated in Figure 1B.

### Data Processing

Data were processed using LabVIEW. Data assessors were blinded so that they were unaware as to which conditions they were processing. Each perturbation was analyzed as a 600 ms interval, comprising 100 ms prior to and 500 ms after perturbation onset.

COP displacement [cm] was recorded for different time intervals, including the starting position right before surface translations (−1 ms, COP_onset_), as well as at 90 ms (COP_90ms_) and 120 ms (COP_120ms_) after the onset of perturbation. Additionally, COP_peak_ was calculated for each perturbation as the difference between COP_onset_ and the peak excursion in the 500 ms window after perturbation onset (Freyler et al., 2015). COP peak index [ms] was assessed for each trial recorded for the 3 protocols by subtracting the index of COP_peak_ from COP_onset_.

Ankle, knee and hip joint kinematics were expressed as changes in joint excursions [Δ°] between onset and peak angle position in the time window of 500 ms after perturbation onset (Freyler et al., 2015). The index of the peak defection was also assessed. Onset joint position was calculated for the ankle, knee, and hip joints [°].

For each of the recorded muscles, EMG signals were rectified and integrated (iEMG [mVs]). For data analysis of trials without PNS, iEMG was divided into four relevant phases before and after perturbation: pre-activation −100 to 0 ms prior to perturbation (PRE), 30–60 ms (SLR; Rinalduzzi et al., 2016), 60–85 ms (MLR), 85–120 ms (LLR; Taube et al., 2006) and 120-peak COP displacement as the belated muscle response (BMR) post perturbation. Subsequently, iEMGs were time normalized [mV/s] and normalized to the MVC [%MVC]. For that purpose, EMG during MVC was integrated for each muscle for a time frame of 1 min [mVs].

Peak-to-peak amplitudes of the H-reflexes and M-waves were calculated [mV].

To compare the different levels of prediction in the tables and figures, all values were normalized to the respective values recorded in protocol (i), in which the platform displacement was correctly predicted.

### Statistics

To test for predictability-induced changes over time, repeated measures analysis of variance (rmANOVA) was used [anticipation (*Predicted* vs. *Unpredicted* vs. *Cheat*)] for the EMGs, H-reflexes, M-waves, joint defections and COP displacements, and their corresponding index, respectively. *Muscle group* (shank vs. thigh vs. hip) was included as a within-subject factor to detect differences between SOL, MG, TA (shank) and RF, BF (thigh), and Gmax (hip). *Phase* was included as a within-subject factor to detect differences between reflex phases SLR, MLR, LLR, and BMR. The normality of the data was evaluated with the Kolmogorov-Smirnov test; data followed a normal distribution. If the assumption of sphericity measured by Mauchly's sphericity test was violated, the Greenhouse-Geisser correction was used. The level of significance was set to *p* < 0.05 and statistically significant differences were marked in data sets with a symbol (^*^). The false discovery rate was controlled according to the Benjamini-Hochberg-Yekutieli method, a less conservative but still stringent statistical approach conceptualizing the rate of type I errors (Benjamini and Yekutieli, 2005). Partial Eta squared ($\eta_{\text{p}}^{2}$) was also used as an estimate of the effect size for the ANOVA ($\eta_{\text{p}}^{2}$ < 0.01 small, 0.1 ≤ $\eta_{\text{p}}^{2}$ ≤ 0.06 medium, 0.24 < $\eta_{\text{p}}^{2}$ large effect size; Cohen 1973). Two-tailed Spearman-Rho correlation analysis was executed to display the relationship between the recorded neuromuscular (EMG and H-reflex amplitudes) and kinematic variables (COP displacement and peak index).

All analyses were executed using IBM SPSS Statistics for Windows, Version 23.0 (IBM Corp., Armonk, NY, USA). Values are presented as mean values ± standard deviations (M ± SD).

## Results

### Postural Sway

**Figure 2:**
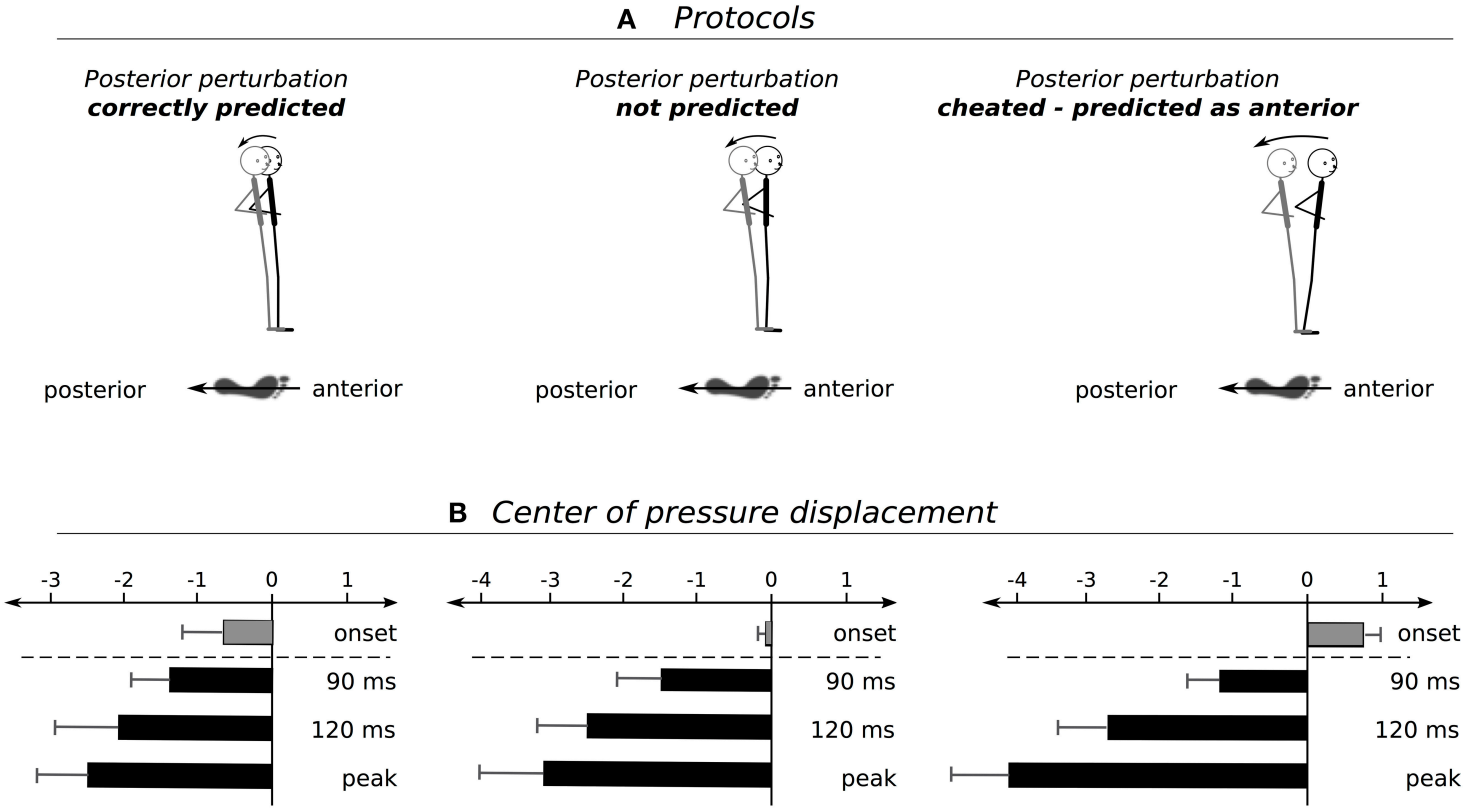
**(A)** Schemes show the postural set at perturbation onset with respect to predicted (left), unpredicted (middle), and cheated (right) perturbations and **(B)** the subsequent changes in body positioning to compensate for the deterioration of posture. Graphs illustrate changes of the center of pressure (COP) displacement at onset prior to the surface translation (gray bars), 90 ms, 120 ms and at the peak displacement after perturbation onset (black bars).

Grand means of COP displacements are presented in Table 1 and Figure 2. The rmANOVA revealed a significant *anticipation x phase* interaction effect. Furthermore, statistically significant distinctions were established for COP_onset_, COP_90ms_, and COP_120ms_ after perturbation as well as for COP_peak_ and its index (Table 1). Results indicate a gradual increase in total COP displacement from *Predicted* to *Unpredicted* to *Cheat* concomitant with a delay of the compensatory COP reaction reflected by an increased peak index. The effect sizes ranged from moderate to high.

### Joint Goniometry

**Figure 3:**
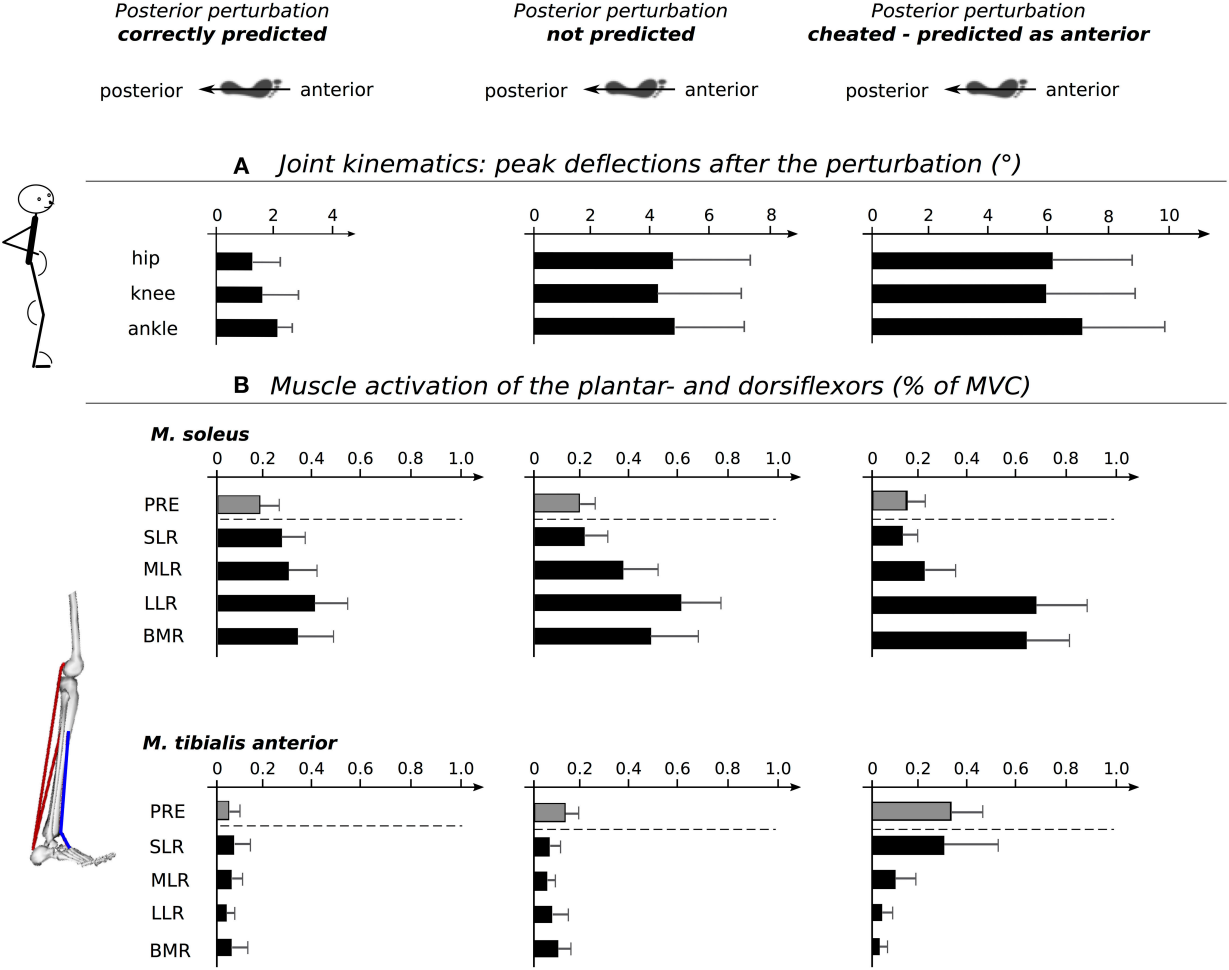
Schemes show the postural set at perturbation onset with respect to predicted (left), unpredicted (middle) and cheated (right) perturbations Below, diagrams showing peak deflections at ankle, knee and hip joints **(A)**. Note that the joint excursions increase from predicted to unpredicted to cheated perturbations. **(B)** Diagrams illustrating the changes in the plantar- and dorsiflexor neuromuscular activation intensities for the distinct time periods before and after perturbation: pre-activation (PRE, gray), short- (SLR), medium- (MLR), and long latency response (LLR), as well as the belated muscle response (BMR, all in black). The dotted line separates the proactive changes prior to perturbation from the reactive changes after the perturbation. Anticipation-induced changes are manifested for neuromuscular control of antagonists encompassing the ankle joint prior and after perturbation. Statistical results are presented in Tables 1, 2.

Grand means of joint deflections are presented in Table 1 and Figure 3. The rmANOVA revealed a significant *anticipation x phase* interaction effect. Ankle, knee, and hip joint excursions increased from *Predicted* to *Unpredicted* to *Cheat*; likewise, the index of the peak deflection increased. No changes were observed for the joint positions at the onset of perturbation. The effect sizes ranged from moderate to high.

### EMG

Modulations in EMG activity are displayed in Table 2 and Figure 3. The rmANOVA revealed a significant *anticipation x phase* interaction effect for SOL, MG, and TA. Furthermore, the rmANOVA revealed a significant *anticipation x muscle group* interaction effect for PRE, MLR, and LLR. The effect sizes ranged from moderate to high.

**Table 2:** Neuromuscular parameters; grand means of EMG integrals during pre-activation (PRE, −100 to 0 ms), the reflex phases SLR, MLR, LLR, and the BMR (120 ms–individual peak of COP) after perturbation, for the muscle groups encompassing the ankle joint (SOL, soleus; MG, medial gastrocnemius; TA, tibialis anterior) are displayed.

**Parameter**			**Protocols**			**Statistics**	
**Neuromuscular activity**		**Phase**	**Predicted**	**Unpredicted**	**Cheated**	***rmANOVA interaction***	***rm*ANOVA**
SHANK	EMG SOL [% of MVC]	PRE **SLR** **MLR** **LLR** **BMR**	0.18 ± 0.08 **0.27 ± 0.10** **0.30 ± 0.12** **0.41 ± 0.14** **0.34 ± 0.15**	0.18 ± 0.06 **0.20 ± 0.09** 0.35 ± 0.14* 0.58 ± 0.16* 0.46 ± 0.19*	0.14 ± 0.07* 0.12 ± 0.06* 0.21 ± 0.12* 0.65 ± 0.20* 0.61 ± 0.17*	***P*** **= 0.041;** ***F*** **= 5.0**	*P* = 0.219; *F* = 0.9; $\eta_{\text{p}}^{2}$ = 0.8 ***P*** **= 0.067;** ***F*** **= 2.1;** $\eta_{\text{p}}^{2}$ **= 0.19** ***P*** **< 0.001;** ***F*** **= 10.3;** $\eta_{\text{p}}^{2}$ **= 0.61** ***P*** **= 0.038;** ***F*** **= 7.7;** $\eta_{\text{p}}^{2}$ **= 0.29** ***P*** **= 0.028;** ***F*** **= 3.9;** $\eta_{\text{p}}^{2}$ **= 0.20**
	EMG MG [% of MVC]	PRE SLR MLR **LLR** **BMR**	0.14 ± 0.09 0.22 ± 0.08 0.25 ± 0.12 **0.33 ± 0.27** **0.37 ± 0.23**	0.12 ± 0.09 0.19 ± 0.07 0.29 ± 0.14 0.43 ± 0.25* 0.45 ± 0.47	0.08 ± 0.04 0.12 ± 0.09 0.21 ± 0.10 0.39 ± 0.21* 0.51 ± 0.26*	***P*** **= 0.021;** ***F*** **= 4.3**	*P* = 0.278; *F* = 0.5; $\eta_{\text{p}}^{2}$ = 0.03 *P* = 0.081; *F* = 0.8; $\eta_{\text{p}}^{2}$ = 0.01 *P* = 0.311; *F* = 1.3; $\eta_{\text{p}}^{2}$ = 0.04 ***P*** **= 0.010;** ***F*** **= 5.5;** $\eta_{\text{p}}^{2}$ **= 0.22** ***P*** **< 0.001;** ***F*** **= 9.0;** $\eta_{\text{p}}^{2}$ **= 0.38**
	EMG TA [% of MVC]	**PRE** **SLR** MLR LLR BMR	**0.05 ± 0.04** **0.07 ± 0.06** 0.06 ± 0.04 0.04 ± 0.03 0.06 ± 0.05	**0.12 ± 0.05** **0.06 ± 0.04** 0.05 ± 0.03 0.07 ± 0.06 0.09 ± 0.05	0.31 ± 0.12* 0.28 ± 0.21* 0.09 ± 0.08 0.04 ± 0.03 0.03 ± 0.03	***P*** **= 0.030;** ***F*** **= 5.9**	***P*** **= 0.004;** ***F*** **= 9.9;** $\eta_{\text{p}}^{2}$ **= 0.41** ***P*** **< 0.001;** ***F*** **= 31.5;** $\eta_{\text{p}}^{2}$ **= 0.55** *P* = 0.229; *F* = 1.2; $\eta_{\text{p}}^{2}$ = 0.07 *P* = 0.311; *F* = 0.9; $\eta_{\text{p}}^{2}$ = 0.03 *P* = 0.824; *F* = 0.7; $\eta_{\text{p}}^{2}$ = 0.05

Data are illustrated for the predicted, unpredicted and cheated protocols. P and F values denote the level of significance of phase x anticipation interaction effects. Bold letters represent a significant main effect for anticipation based on rmANOVA. ^*^indicates significant differences compared to the predicted condition.

TA showed a significant increase in PRE and SLR from *Predicted* to *Unpredicted* to *Cheat* indicating differences in the muscle pre-set prior to perturbation (Table 2). After perturbation, SOL, MG, BF, and GMax were significantly affected: SOL, MG, and BF EMG activity increased significantly in the LLR and BMR from *Predicted* to *Unpredicted* to *Cheat*. Additionally, SOL EMG activity was augmented for SLR and MLR from *Predicted* to *Unpredicted* to *Cheat* (Table 2). For GMax, EMG activity increased in the BMR from *Predicted* to *Unpredicted* to *Cheat*. The effect sizes ranged from moderate to high.

### H-Reflexes

**Figure 4:**
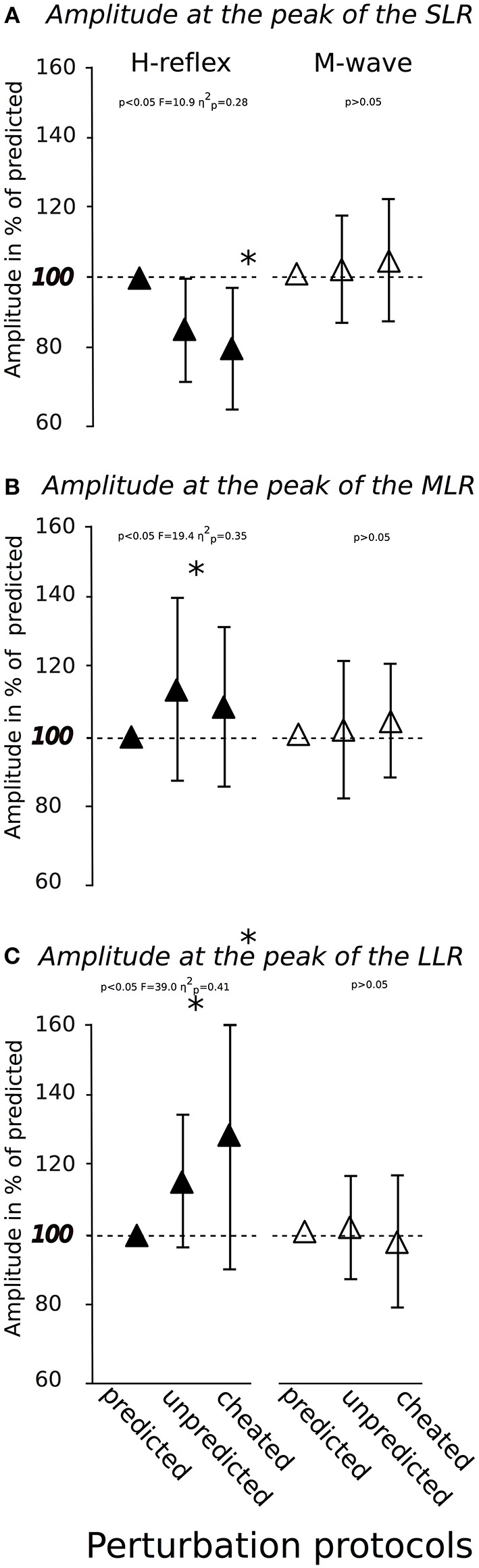
Modulation of soleus H-reflexes (▴left) and M-waves (Δ right) in response to predicted, unpredicted and cheated perturbations (x-axis) provoked at the peak of the short- **(A)**, medium- **(B)**, and long-latency response **(C)**. The horizontal dashed red line marks the initial value recorded in the control condition of predicted perturbations.*indicates significant differences compared to the control condition.

Modulations in H-Reflex and M-wave amplitudes are displayed in Figure 4. The rmANOVA revealed a significant *anticipation x phase* interaction effect for the H-reflex amplitude in the SLR (Figure 4A), MLR (Figure 4B) and LLR (Figure 4C), indicating a reduction in spinal excitability in SLR from *Predicted* to *Unpredicted* to *Cheated* and an increased spinal excitability in the LLR from *Predicted* to *Unpredicted* to *Cheated*. M-wave amplitudes and SOL activity during PRE remained unchanged. The effect sizes ranged from moderate to high.

### Correlations

**Figure 5:**
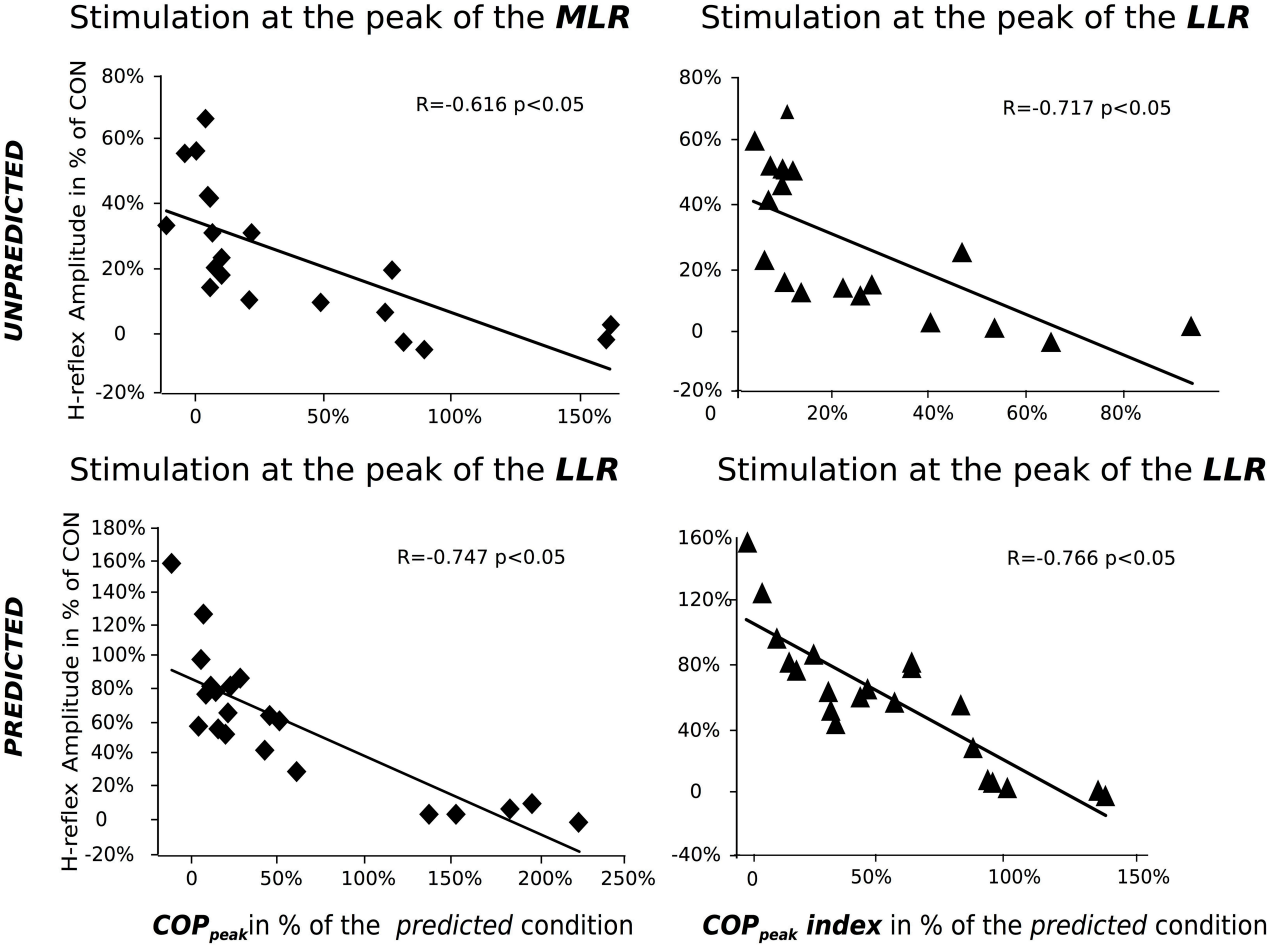
Bivariate correlations among the variables center of pressure (COP), displacement (abscissa), and H-reflex amplitude (ordinate) for the unpredicted (top) and predicted (bottom) perturbation conditions. Graphs illustrate the interrelationship of COP_peak_ (left) and COP_peak_ index (right) with the Soleus H-reflex amplitudes elicited at the peak of the MLR (top left) and LLR (top right and bottom), respectively. Correlation coefficients and regression lines indicate a negative relationship between the neuromuscular and kinematic variables, with almost linear muscle- and phase-specific associations.

Bivariate correlation coefficients *R* and *p*-values for H-reflexes, SLR, MLR, and LLR are displayed in Table 4 and Figure 5. For the *predicted conditions*, a negative correlation was detected between COP_onset_ and COP_peak_ (*R* = −0.699, *p* = 0.001), as well as COP_peak_ and SOL PRE (*R* = −0.523, *p* = 0.040).

For the *Unpredicted* conditions with randomly applied perturbations, significant negative correlations were detected between COP_peak_ and the SOL H-reflex amplitude in the MLR, SOL EMG in the MLR and LLR and MG EMG in the LLR and BMR. Furthermore, COP_peak_ index was negatively correlated to H-reflex amplitudes in LLR. BF EMG activity in LLR was positively correlated with the knee joint excursion (*R* = 0.501, *p* = 0.044).

For *cheated* conditions containing wrongly predicted perturbations, we detected a significant positive correlation between COP_onset_ and COP_peak_ (*R* = 0.771, *p* = 0.002) as well as COP_peak_ and TA PRE (*R* = 0.601, *p* = 0.025). Furthermore, we detected a significant negative correlation between COP_peak_ and the H-reflex amplitude in LLR (Figure 4), the EMG in LLR for SOL and MG, as well as BMR for GMax, respectively (Table 4). COP_peak_ index was negatively correlated to H-reflex amplitudes in LLR (Figure 4) and EMG of SOL and MG at LLR, as well as BMR for MG, SOL, and GMax (Table 4). BF EMG activity in LLR was positively correlated with knee joint excursion (*R* = 0.647, *p* = 0.004).

## Discussion

The current study advances our understanding of the mechanisms underlying postural reactions to imposed perturbations. Modulations in response to stimulus prediction were phase- and segment-specific and associated with differences in the balance strategy when the postural set was manipulated by providing either (i) *predictable*, (ii) *unpredictable*, or (iii) *cheated* perturbations. From (i) to (iii), results demonstrated progressively: (a) increased spinal excitability in the MLR and LLR; (b) delayed muscle activities; (c) shifted activation patterns, with muscles encompassing the proximal segment being more involved in the compensatory postural response; and (d) increased COP displacements and angular excursions of the ankle, knee, and hip joints. Neuromechanical coupling was manifested by positive correlations for the anticipation-induced changes in EMG activity and spinal excitably with the displacement and timing of the COP trajectories.

Two aspects might be important in interpreting these findings. The first one deals with proactive postural adjustments *prior* to perturbations and the second one with compensatory postural reactions *after* perturbations (Horak and Nashner, 1986; Horak et al., 1989; Mohapatra et al., 2012).

### Proactive Modulations Associated With Anticipatory Postural Adjustments

Analysis of predicted, in contrast to unpredicted, perturbations revealed significant proactive events prior to perturbation. Proactivity was manifested by a backwards lean when perturbations were correctly forecasted to occur in a posterior direction. This contrasted with a *neutral* positioning at a centrally orientated trajectory when perturbation was initiated without auditory pre-cueing of the movement direction (Figure 2). These pre-adjustments are governed by supraspinal structures of the human brain (Taube et al., 2006, 2007). In agreement with previous investigations (Jacobs et al., 2008; Kanekar and Aruin, 2014a,b; Welch and Ting, 2014), the outcomes showed that prior information allowed subjects to prepare for an impending stimulus by slightly shifting body posture toward an advantageous pre-set position (Jacobs et al., 2008; Kanekar and Aruin, 2014a,b). The shift was considered advantageous insofar, as the resulting COM displacement could be diminished in comparison to neutral positioning (Figure 2). Correlations furthermore demonstrated that the higher the SOL PRE, the smaller the COP_peak_, indicating that the CNS used prior knowledge to select and activate beneficial muscles adequately prior to stimulus onset. Therefore, deteriorations of posture could be alleviated in advance. Importantly, both pre-activation and angular deflections are associated with neuromechanical coupling to adapt muscle length, including pre-stretch (Houk et al., 1970; Polus et al., 1991) and length-tension characteristics that affect the passive muscle forces (Houk et al., 1970) and determine the subsequent compensatory reflex responses in the musculature counteracting the perturbation stimulus (Polus et al., 1991).

When subjects were cheated with the expectation of anterior instead of posterior movement of the support surface, we observed opposed modifications in the *proactive postural set*. Instead of a backwards lean, an anterior COP shift occurred concomitant with augmented TA pre-activity (Figure 3). Correlations indicated that the more the subjects activated TA and leaned forward to prepare for the expected anterior perturbation, the higher the maximal COP excursion in a posterior direction upon the surprising backward perturbation, and the greater the postural instability (Pater et al., 2015). Thus, it became apparent that forewarning using valid information allowed individuals to anticipate and so, pre-adjust body segments appropriately according to the impending stimulus characteristics (Jacobs et al., 2008; Mochizuki et al., 2008). However, Invalid information led to an overestimation and subsequent muscle preset to compensate for the severe postural deficits exposed by the mechanical stimulus.

### Reactive Modulations Associated With Compensatory Postural Adjustments

Beyond the proactive preset, compensatory mechanisms following the perturbation have been identified as *reactive adjustments* to prevent from falling and to counteract the deterioration of postural equilibrium (Horak and Nashner, 1986; Gollhofer and Rapp, 1993). These include phasic and segmental distinctions of neuromuscular activation: *Phasic distinctions* refer to the timing and chronology of neuromuscular modulations associated with kinematic adaptations. Our findings showed the importance of the late poly-synaptic reflexes (Gollhofer et al., 1989) in compensating for the unpredicted postural disturbances (concerning MLR, LLR, and BMR), and even more so for the cheated ones (concerning LLR and BMR). With reference to Santos et al. (2010) and Welch and Ting (2014), neuromuscular activity was distinctly elevated in LLR and BMR concomitant with increased joint excursions and their indices for both protocols (Table 1). The functional relevance was supported by the negative correlations between late EMG responses in the thigh and shank musculature and COP displacements. These indicated that the earlier the support surface translations could be counterbalanced and the higher the EMG boost, the smaller was the magnitude of postural sway and the faster the individual recovered postural equilibrium (Table 4).

To gain further insight into the modulating processes underlying the adaptations associated with the stimulus anticipation, SOL H-reflex were elicited at the peak of the SLR, MLR, and LLR for an assessment of the Ia afferent muscle spindle input to the neuromuscular response (Taube et al., 2008). Results revealed facilitated H-reflexes for MLR and LLR in the anticipation of unpredicted perturbations (Figure 4). This demonstrated the pivotal role of spinal facilitation in light of stimulus anticipation, which was further emphasized by the negative correlations between H-reflex amplitudes at MLR and LLR with the COP_peak_ and its corresponding index. With reference to Dietz et al. (1991) and Gollhofer et al. (1989), who also showed that the critical time point for reflex responses of functional importance was > 60 ms, though without considering the predictability of postural sets, we concluded that uncertainty about the upcoming perturbation stimulus could be counterbalanced by the CNS using long-loop polysynaptic reflexes. These reflexes have functional significance in inducing appropriate joint moments to maintain upright posture (Horak and Nashner, 1986; Gollhofer et al., 1989; Horak et al., 1989) under the governance of the brains's descending drive (Taube et al., 2006, 2007).

In consideration of the results obtained in the cheated condition, the aspect of *specificity of prediction* should be considered. Interestingly, the H-reflex was facilitated in the LLR, but unaffected in MLR and even diminished in SLR (Figure 4). Hence, the phase of functional importance was shifted by 30 ms, and this delay differed considerably from both predicted and unpredicted conditions (Gollhofer et al., 1989; Dietz et al., 1991). Thus, the belated kinematic compensation reflected by a delayed and increased COP excursion was not surprising (Table 1). Notably, SOL H-reflexes were still inhibited in SLR for cheated conditions when an anterior perturbation was forecast. With reference to Jacobs and colleagues (Jacobs et al., 2008), we concluded that error detection did not happen in SLR, although it would be highly advantageous if the SOL spindle reflex boosted the EMG to provoke an immediate muscle contraction, to compensate for the sudden posterior surface translation (Gollhofer et al., 1989; Gollhofer and Rapp, 1993). This failure was only corrected in LLR. Therefore, the anticipatory pre-setting was still valid in the early reflex phase (SLR). On the basis of recent studies (Taube et al., 2008; Tokuno et al., 2009), we assumed that a beneficial adjustment of spinal excitability under the governance of supraspinal centers due to incorrect stimulus prediction could be achieved 90 ms after perturbation onset, but not before.

It is worthwhile to discuss the *segmental distinction* concomitant with aforementioned neuromuscular modulations. Comparing the three modalities with progressively increasing difficulty (Horak et al., 1989), it was apparent that diminished information about the upcoming perturbation, or unreliable stimulus prediction, induced a multi-segmental strategy involving the proximal segment in the compensatory postural response (Tables 1–3). Confirmed by the interrelation of GMax and COP_peak_ (Table 4) in the cheated protocol, the increased neuromuscular control of musculature proximal to the body's COM enabled control of the trunk, emphasizing a quick reacquisition of postural control after external disturbances provoking falls (Horak and Nashner, 1986). Additionally, the increased BF activity occurred concomitantly with an augmented knee flexion (Table 1) associated with a lowering of the COM in the vertical plane (Bhatt et al., 2006; Di Giulio et al., 2013). The decrease of the COM height allowed a rapid reacquisition of a stable COM position during unpredictable slips. This is also known to be essential for posture safety and reduction of fall risk (Bhatt et al., 2006). In consideration of muscle topography and the associated balance strategy (Horak and Nashner, 1986), it is suggested that a multi-segmental reaction is the preferred strategy to relocate the COM back to a stable area above the feet if stimulus anticipation is limited or impossible (Horak and Nashner, 1986; Maki and McIlroy, 1996).

**Table 3:** Neuromuscular parameters; grand means of EMG integrals during pre-activation (PRE, −100 to 0 ms), the reflex phases SLR, MLR, LLR, and the BMR (120 ms–individual peak of COP) after perturbation, for the muscle groups encompassing the hip (gluteus maximus, GMax) and knee (RF, rectus femoris; BF, biceps femoris) are displayed.

**Parameter**			**Protocols**			**Statistics**	
**Neuromuscular activity**		**Phase**	**Predicted**	**Unpredicted**	**Cheated**	***rmANOVA interaction***	***rm*ANOVA**
THIGH	EMG GMax [% of MVC]	PRE SLR MLR LLR **BMR**	0.21 ± 0.09 0.18 ± 0.07 0.23 ± 0.10 0.20 ± 0.11 **0.26 ± 0.13**	0.22 ± 0.08 0.20 ± 0.08 0.21 ± 0.10 0.22 ± 0.09 **0.31 ± 0.17**	0.19 ± 0.11 0.18 ± 0.07 0.24 ± 0.12 0.27 ± 0.11* 0.40 ± 0.15*	*P* = 0.501; *F* = 0.9	*P* = 0.553; *F* = 1.4; $\eta_{\text{p}}^{2}$ = 0.06 *P* = 0.251; *F* = 0.4; $\eta_{\text{p}}^{2}$ = 0.07 *P* = 0.784; *F* = 0.9; $\eta_{\text{p}}^{2}$ = 0.02 *P* = 0.913; *F* = 0.1; $\eta_{\text{p}}^{2}$ = 0.01 ***P*** **< 0.001;** ***F*** **= 15.8;** $\eta_{\text{p}}^{2}$ **= 0.27**
	EMG RF [% of MVC]	PRE SLR MLR LLR BMR	0.12 ± 0.07 0.13 ± 0.09 0.10 ± 0.11 0.14 ± 0.07 0.17 ± 0.11	1.04 ± 0.67 1.04 ± 0.69 1.04 ± 0.68 1.02 ± 0.65 0.15 ± 0.09	1.00 ± 0.65 1.07 ± 0.84 0.97 ± 0.66 1.06 ± 0.64 0.19 ± 0.13	*P* = 0.711; *F* = 0.3	*P* = 0.903; *F* = 0.1; $\eta_{\text{p}}^{2}$ = 0.01 *P* = 0.447; *F* = 0.6; $\eta_{\text{p}}^{2}$ = 0.06 *P* = 0.691; *F* = 1.3; $\eta_{\text{p}}^{2}$ = 0.06 *P* = 0.340; *F* = 0.9; $\eta_{\text{p}}^{2}$ = 0.08 *P* = 0.217; *F* = 0.5; $\eta_{\text{p}}^{2}$ = 0.04
	EMG BF [% of MVC]	PRE SLR MLR **LLR BMR**	0.12 ± 0.08 0.14 ± 0.10 0.16 ± 0.09 **0.20** ± **0.12** **0.23 ± 0.13**	0.10 ± 0.05 0.11 ± 0.07 0.18 ± 0.12 **0.25 ± 0.16** 0.28 ± 0.12*	0.12 ± 0.09 0.10 ± 0.10 0.15 ± 0.11 0.34 ± 0.17* 0.39 ± 0.19*	*P* = 0.182; F = 1.0	*P* = 0.333*; F* = 0.6; $\eta_{\text{p}}^{2}$ = 0.04 *P* = 0.541; *F* = 0.9; $\eta_{\text{p}}^{2}$ = 0.02 *P* = 0.711; *F* = 0.4; $\eta_{\text{p}}^{2}$ = 0.03 ***P*** **= 0.018;** ***F*** **= 7.3;** $\eta_{\text{p}}^{2}$ **= 0.45** **P < 0.001; F = 9.9;** $\eta_{\text{p}}^{2}$ **= 0.83**

P and F values denote the level of significance of phase x anticipation interaction effects. Bold letters represent a significant main effect for anticipation based on rmANOVA. ^*^indicates significant differences compared to the predicted condition.

**Table 4:** Bivariate Spearman-Rho correlation coefficients (CC), *R* and *p*-values among the variables COP_peak_ and COP_peak_ index with the normalized EMGs of GMax, BF, SOL, MG and TA in the relevant phases before and after perturbation: PRE (100 ms before perturbation), SLR (30–60 ms after perturbation onset), MLR (60–85 ms after perturbation onset), LLR (85–120 ms after perturbation onset) and BMR (120 ms–peak COP displacement).

		**Protocols**			
		**Unpredicted**		**Cheated**	
		**COP_**peak**_ [% of predicted]**	**COP_**peak**_ index [% of predicted]**	**COP_**peak**_ [% of predicted]**	**COP_**peak**_ index [% of predicted]**
**Neuromuscular parameters**	**PHASE**	**CC *R, p***	**CC *R, p***	**CC *R, p***	**CC *R, p***
H-reflex amplitude	MLR LLR	***R*** **= −0.616**, ***p*** **= 0.003** *R* = −0.386, *p* = 0.093	*R* = −0.198, *p* = 0.403 ***R*** **= −0.717**, ***p*** **= 0.019**	*R* = −0.657, *p* = 0.062 ***R*** **= −0.747**, ***p*** **< 0.001**	*R* = −0.320, *p* = 0.157 ***R*** **= −0.766**, ***p*** **< 0.001**
EMG GMax[% of predicted]	BMR	*R* = – 0.100, *p* = 0.749	*R* = 0.028 *p* = 0.684	***R*** **= 0.620**, ***p*** **= 0.002**	***R*** **= −0.692**, ***p*** **< 0.001**
EMG SOL[% of predicted]	MLR LLR	**R = −0.676**, ***p*** **= 0.001** ***R*** **= −0.610**, ***p*** **= 0.003**	R = 0.072, *p* = 0.761 *R* = −0.299, p = 0.177	R = −0.291, *p* = 0.659 ***R*** **= −0.622**, ***p*** **= 0.004**	**R = −0.816**, ***p*** **< 0.001** ***R*** **= −0.576**, ***p*** **= 0.006**
EMG MG[% of predicted]	LLR BMR	***R*** **= −0.715**, ***p*** **< 0.001** ***R*** **= 0.877**, ***p*** **< 0.001**	*R* = −0.311, *p* = 0.083 *R* = −0.050, *p* = 0.834	***R*** **= −0.589**, ***p*** **= 0.006** *R* = −0.716, *p* < 0.001	***R*** **= 0.576**, ***p*** **= 0.006** ***R*** **= −0.703**, ***p*** **< 0.001**

*Only significant findings are shown. Findings revealed muscle- and phase-specific distinctions. Values are presented as a percentage of the value during predicted perturbations (CON). Bold values indicate statistical differences*.

## Limitation

For a conclusive statement, it is crucial to consider the limitations of the study. Thereby, the choice of subjects is of substantial importance. The volunteers were a homogeneous sample of young and healthy participants. However, they were not subdivided into athletes and non-athletes although the level of sportiness and training experience may be related to different characteristics in balance skills (Vuillerme et al., 2001). Therefore, the results of this study do neither reflect inter-individual differences nor allow to distinguish between particular sub-populations.

## Conclusion and Perspectives

These findings advance our understanding about the neuromechanical mechanisms underlying postural reactions to imposed perturbations. Modulations in response to systematic changes in stimulus prediction were phase- and segment-specific, and were associated with differences in the neuronal and kinematic balance strategy when the postural set was modulated using either predictable, unpredictable, or cheated perturbations. A shift in multi-segmental organization involving proximal muscle groups, and facilitated late reflex responses compensating for cheated or unpredictable perturbations, have been shown to achieve balance recovery and restore a safe body equilibrium. Despite this phasic and segmental distinction, a clear difference in regard to the proactive set was found, relying on muscle pre-activation and body positioning. With an emphasis on the CNS, the results demonstrate the importance of considering the context under which recovery responses are assessed.

Beyond a basic understanding about posture control, the outcomes can support prevention programs during which task-specific perturbation training is administered. Thereby, postural sets involving correctly predicted responses can serve as an adequate exercise regime with the focus on the descending drive of the CNS to control postural equilibrium. This may evolve an adequate pre-program of muscle activation in the lower limb to counteract a specific type of postural disturbance. With an emphasis on stimulus anticipation and a certain flexibility of the postural responses, postural control rehabilitation may implement unpredicted or cheated perturbations. These settings may focus on immediate and long-loop reflexes and may lead to learning effects associated with an improved reflectory control after postural deteriorations that may be of significance for specific patient populations or athletes (Krause et al., 2018).

## Author Contributions

RR and KF: Conceptualization; RR, KL, AK, AG and KF: Investigation; RR, KL, AG, and KF: Methodology; RR and AG: Project administration; RR and AG: Resources; AG: Supervision; RR: Software, writing of the original draft, figure and tables; RR, KL, AK, AG, and KF: Review and editing.

### Conflict of Interest Statement

The authors declare that the research was conducted in the absence of any commercial or financial relationships that could be construed as a potential conflict of interest.
